# Metabolic influences on T cell in psoriasis: a literature review

**DOI:** 10.3389/fimmu.2023.1279846

**Published:** 2023-11-15

**Authors:** Rina Su, Siqi Zhao, Jinqing Zhang, Mei Cao, Shiguang Peng

**Affiliations:** Department of Dermatology, Beijing Chao-yang Hospital, Capital Medical University, Beijing, China

**Keywords:** glucose metabolism, lipid metabolism, amino acid metabolism, psoriasis, T cell

## Abstract

Psoriasis is a systemic inflammatory disease that frequently coexists with various other conditions, such as essential hypertension, diabetes, metabolic syndrome, and inflammatory bowel disease. The association between these diseases may be attributed to shared inflammatory pathways and abnormal immunomodulatory mechanisms. Furthermore, metabolites also play a regulatory role in the function of different immune cells involved in psoriasis pathogenesis, particularly T lymphocytes. In this review, we have summarized the current research progress on T cell metabolism in psoriasis, encompassing the regulation of metabolites in glucose metabolism, lipid metabolism, amino acid metabolism, and other pathways within T cells affected by psoriasis. We will also explore the interaction and mechanism between psoriatic metabolites and immune cells. Moreover, we further discussed the research progress of metabolomics in psoriasis to gain a deeper understanding of its pathogenesis and identify potential new therapeutic targets through identification of metabolic biomarkers associated with this condition.

## 1 Introduction

Psoriasis, a chronic dermatological ailment known for its persistent inflammatory nature, affects nearly 3% of individuals worldwide (1). While the roots of this condition are multifaceted, spanning genetic factors, environmental influences, lifestyle habits, and more, the crux lies in the disruption of the immune system’s equilibrium (2). As individuals navigate through the stages of psoriasis, they often encounter an array of secondary health challenges. These associated ailments include, but are not limited to, psoriatic arthritis, cardiovascular issues, metabolic disorders such as diabetes, non-alcoholic hepatic steatosis, and inflammatory conditions affecting the gastrointestinal tract (3). Fresh research findings have begun to highlight an unsettling inclination among psoriasis patients: a deviation in lipid metabolism. Such a deviation casts a direct shadow on conditions like metabolic syndrome and cardiovascular complications (4). This skewed metabolic activity amplifies the risk factors associated with grave events such as myocardial infarctions and thromboembolisms, casting a pall over patient longevity (5, 6). Gaining an in-depth grasp of the intertwined pathophysiological underpinnings that connect psoriasis-driven inflammation to these metabolic perturbations is indispensable. This knowledge not only offers a clearer lens to view the disease but also unravels the nuanced nexus between the primary condition and its myriad comorbidities.

The intricate etiological framework of psoriasis recognizes a myriad of triggers, notably physical traumas, microbial invasions, and drug-related repercussions. When such events occur, they lead to the release of endogenous nucleotides which subsequently engage with antimicrobial peptides (AMPs). Through this partnership, receptors such as Toll-like receptor 7 (TLR7) and TLR9 on plasmacytoid dendritic cells (pDCs) get activated. Once aroused, pDCs unleash a generous surge of interferon-alpha (IFN-α), setting the stage for cutaneous myeloid dendritic cell (mDC) activation. These mDCs then transition into their mature state, becoming a prolific source of pivotal cytokines: IL-6, IL-12, IL-23, and TNF-α. This unique cytokine blend steers initial T cell differentiation towards Th1, Th17, and Th22 lineages, with a pronounced emphasis on Th17 lineage augmentation. The intricate dance of signaling molecules, encompassing IL-6, TGFβ, IL-23, and IL-1β, meticulously orchestrates the Th17 cell differentiation process. As Th17 cells mature, they not only express RORγt and RORα but also become potent sources of proinflammatory agents such as IL-17A and IL-17F. The latter are especially detrimental to keratinocytes, causing their unchecked proliferation and skewed differentiation patterns. These deviant keratinocytes further intensify the situation by churning out additional chemokines and adhesive entities, thus magnetizing neutrophils and sparking an incessant loop of immune-inflammatory reactions (7). It’s pivotal to understand that this T-cell-mediated inflammatory cascade lies at the crux of psoriatic onset and persistence.

Within the intricate framework of psoriasis pathophysiology, a noteworthy metabolic alteration is observed in T cells. Instead of conventionally relying on glucose breakdown and the decomposition of fatty acids and amino acids, they predominantly shift focus to aerobic glycolysis complemented by anabolic processes. Such a metabolic recalibration is more than just an energy-generating mechanism for ATP synthesis. It goes beyond to accumulate vital metabolic intermediates, instrumental in guiding T cell differentiation pathways. It’s fascinating to note how the specific functionalities attributed to diverse T-helper cell lineages are deeply interwoven with foundational metabolic pathways. These encompass glycolytic activity, the process of fatty acid β-oxidation (FAO), lipid synthesis, and amino acid cycling. Through this exposition, our objective is to shed light on the intricate web of interactions between these metabolites and their governing metabolic pathways in influencing T cell dynamics, specifically within the milieu of psoriasis. By doing so, we aspire to not only enrich the understanding of the disease’s origins but also pinpoint promising therapeutic avenues.

## 2 Regulation of glucose metabolism on T cell in psoriasis

Kicking off the glycolytic sequence, cells incorporate glucose from their external environment. Within the cellular cytoplasm, glucose undergoes rapid transformation into pyruvate, alongside generating various intermediary metabolites. Significantly, this swift process circumvents mitochondrial involvement. Under the influence of strong inducers, notably growth factors, the uptake rate of glucose and the pace of the glycolytic progression see a marked amplification. This acceleration culminates in the augmented synthesis of biosynthetic intermediates, paramount for cells undergoing brisk division. Specific subsets of activated effector T cells, with Th17 cells (8) standing out prominently, display a heightened glycolytic footprint. This metabolic trend also extends to Th1, Th2, and CD8+ effector T cells. As such, a spike in glycolytic flux emerges as a characteristic metabolic hallmark across a spectrum of immune-responsive cells. As is well known, therapy targeting activated T cells has achieved great success in the treatment of various diseases. For example, treatment targeting Th17 cells plays an important role in a variety of metabolic diseases other than psoriasis, such as metabolic syndrome, obesity, type 2 diabetes and fatty liver associated with metabolic dysfunction (9, 10). Diving deeper into the intertwined relationship between glycolysis and the development, as well as the multifaceted roles of Th17 cells, might pave the way for innovative metabolic-centric therapeutic interventions targeting psoriasis.

### 2.1 Glycolytic metabolic pathway

Glycolysis is pivotal in the metabolic reconfiguration of immunological cells. Quiescent T cells predominantly depend on oxidative phosphorylation and fatty acid catabolism for their energy needs (11). Upon activation through TCR signaling, these naive T cells switch to a glycolytic metabolic profile to support their differentiation into effector T cells. The subsets of Th1, Th2, and Th17 cells have been observed to rely chiefly on aerobic glycolysis for their operations. Augmenting Glucose transporter 1(Glut1) expression and glucose uptake can potentially amplify their functional capacities. In contrast, Treg cells demonstrate a preference for oxidative phosphorylation and fatty acid breakdown pathways. Foxp3, which is highly expressed in Treg cells, can inhibit glycolysis related genes, and induce gene expression related to lipid and oxidative metabolism (11, 12). B-cell CLL/lymphoma 6 (Bcl-6), as the main transcription factor for Tfh differentiation, can directly inhibit gene expression of various factors in the glycolysis pathway by competing with IL-2 signaling (13).The metabolic characteristics of these cells are closely aligned with their phenotypic attributes, hinting at the possibility of manipulating T cell metabolism as a novel therapeutic strategy for inflammatory conditions (14). Animal studies have shown that the selective small-molecule inhibitor 3-(3-pyridinyl)-1-(4-pyridinyl)-2-propen-1-one (3PO), targeting the key glycolytic enzyme Phosphofructokinase-2/fructose-2,6-bisphosphatase 3 (PFKFB3), disrupts T cell metabolism, culminating in reduced cell proliferation and heightened cell death rates. This disruption has demonstrated efficacy in mitigating psoriasis-like skin conditions in murine models by decreasing CD3+ T cell infiltration in cutaneous lesions (15).

Upon activation, T cells rapidly enhance their glycolytic machinery, facilitated by a diverse ensemble of enzymes and proteins. This cellular metabolic adaptation, central to the transformation journey of T cells as they evolve into their specific subtypes, is meticulously orchestrated by a complex tapestry of transcriptional regulators and signaling pathways. To elucidate, Th1 cell differentiation leans on the regulatory influence of elements such as mTORC1, PI3K, P70S6K, HIF-1α, and Bcl-6. When spotlighting Th17 cells, the regulatory supremacy of mTORC1 and HIF-1α becomes evident. Conversely, the metabolic journey of Treg cells carves a distinctive trail, with AMPK reigning as the chief metabolic maestro (16).

In T cells, the mTOR signaling axis serves as a regulatory hub, influencing various cellular mechanisms such as proliferation, programmed cell death, differentiation, and metabolic processes via activation of downstream transcriptional elements. One notable transcription factor activated by mTOR is Myc, which upregulates the expression of enzymes crucial for aerobic glycolysis and other anabolic metabolic routes. Additionally, mTOR promotes the translation of HIF-1α mRNA. This in turn elevates HIF-1α levels, which act to enhance the expression of glycolytic enzymes and sustain a balance between Treg and pro-inflammatory Th17 cells by facilitating RORγt-mediated transcriptional activation (8, 17). A specific glucose epimer, D-mannose, has been shown to inhibit glycolysis and downregulate HIF-1α, thus mitigating both local and systemic inflammation via suppression mediated by the AKT/mTOR/HIF-1α signaling cascade (18). Intriguingly, the proinflammatory cytokine IL-23 has been identified as a stimulant for CDK7 expression in CD4+ T cells, further amplifying glycolytic activity through activation of the PKB/mTOR/HIF-1α signaling network in a psoriasis-like mouse model induced by imiquimod (IMQ) (19).

Furthermore, the PI3K signaling axis becomes active within CD4+ T cells during cell division or subsequent to TCR complex engagement. This activation triggers the mobilization of Glut1 transporters from the cellular cytoplasm to the plasma membrane, leading to an elevation in glucose uptake and a consequent boost in glycolytic activity (20). A rise in glucose concentrations markedly promotes Th17 cell differentiation, a process that involves an upregulation of intracellular reactive oxygen species (ROS) and subsequent activation of TGF-β signaling pathways. Particularly, mitochondrial ROS (mtROS) are instrumental in the facilitation of TGF-β activation triggered by elevated glucose levels, thereby contributing to the genesis of Th17 cells (21). These observations underscore the potential utility of immuno-metabolic interventions as promising therapeutic strategies for conditions characterized by skin hyperproliferation.

### 2.2 Glucose transporter

Glucose stands as an indispensable cellular resource, especially for rapidly proliferating cells. Its assimilation from the surrounding environment is facilitated by a trio of specialized transport protein families. The primary group belongs to the major facilitator superfamily (MFS) and is commonly identified as GLUTs or SLC2. In parallel, sodium-driven transporters, termed SGLTs, come under the SLC5 category. Additionally, the SWEET family, cataloged as SLC50, plays its part (22). Among the GLUT variants, humanity boasts 14 distinguished members. Delving into their specifics, GLUT1, GLUT3, GLUT4, and GLUT6 primarily act as non-concentrative glucose couriers. On the other hand, for lymphocytes, the task of glucose assimilation rests upon the sodium-dependent SGLT1 (23).

Glut1 demonstrates predominant expression levels across multiple tissues and is central to regulating basal glucose absorption (24). Its role in psoriasis progression is likely due to its influence on excessive keratinization, inflammatory response, and angiogenic processes. A comparative immunohistochemical evaluation has shown an augmented expression, both at protein and mRNA levels, of Glut1 in psoriatic lesions as opposed to non-affected skin and normal controls. Additionally, a significant positive association has been observed between heightened Glut1 levels and metrics such as the psoriasis area severity index (PASI), average thickness of the epidermis, density of inflammatory cells, and microvascular concentration (25). The cutaneous application of glycolysis inhibitor WZB117 was shown to markedly reduce leukocyte (CD45+), neutrophil (Gr-1+), macrophage (F4/80+), and T cell (CD4+) populations in IMQ-treated skin (26). Furthermore, the targeted deletion of Glut1 in T cells exacerbates the characteristics of mouse models exhibiting psoriasis-like skin abnormalities (27).

Previous investigations have elucidated the pivotal influence of the GLUT3 in the functioning of Th17 cells. GLUT3-facilitated glucose entry into these cells governs their metabolic transcriptional patterns at a molecular scope. In-depth analyses using metabolomics, epigenetics, and transcriptomics have uncovered GLUT3 as a key determinant in the epigenetic modulation of inflammatory gene profiles. This transporter is intricately tied to mitochondrial glucose metabolism and the generation of acetyl-CoA through ATP-citrate lyase (ACLY). Blocking the GLUT3-mediated acetyl-CoA synthesis acts as a metabolic safeguard in models of autoimmune colitis and encephalomyelitis, mitigating the inflammatory actions propagated by Th17 cells (28). Given the crucial role of Th17 cell-mediated immune inflammation in the progression of psoriasis, we speculate that GLUT3 may participate in the pathogenesis of psoriasis by regulating the aerobic glycolysis of Th17 cells, and further experiments are needed to verify this possibility.

## 3 Regulation of lipid metabolism on T cell in psoriasis

Lipids, a broad term encompassing both fats and lipoids, are integral to various cellular functions. While fats are essentially triglycerides, lipoids encompass an array of compounds, including steroidal esters, phospholipids, and glycolipids. These lipid derivatives play indispensable roles in effector T cells, serving as key players in cell signaling, energy balance, and protein transportation. A shift in the phospholipid profile within psoriatic lesions implies a role for lipid accumulation in the reticuloendothelial system, contributing to cutaneous inflammation and aberrant keratinization. In the context of psoriasis, pathways linked to lipogenesis and cholesterol synthesis appear to directly impact the differentiation and functionality of Th17 cells. Elevated lipid levels and associated inflammatory processes are established as salient risk elements for both the initiation and recurrence of psoriasis (29). Effective regulation of lipid metabolic processes is crucial for the differentiation of specific Th cell subtypes (30).

A comprehensive meta-analysis has pointed out noteworthy alterations in lipid profiles among psoriasis patients in contrast to a healthy control cohort. Specifically, these patients exhibited elevated concentrations of several lipids, including total cholesterol, triglycerides (TG), low-density lipoprotein (LDL), very low-density lipoprotein (VLDL), lipoprotein(a) (Lp[a]), and apolipoprotein B (Apo B). On the flip side, high-density lipoprotein (HDL) concentrations were found to be markedly reduced (31). Furthermore, metabolomic assessments have disclosed that individuals with psoriasis who also have coexisting metabolic conditions showed a substantial increase in 17 specific metabolites, with ceramide and phosphatidylcholine being especially pronounced. These observations collectively underscore the likely role of lipid metabolic imbalance in the advancement of psoriasis (32).

### 3.1 Fatty acid

Fatty acids have a basic structural formula represented as CH3(CH2)nCOOH. These molecular entities can be categorized in various ways: by the length of their carbon chain, by the degree to which they are saturated, and by the geometry of their double bonds (cis or trans). When discussing carbon chain length, fatty acids with 2-6 carbon atoms are termed ‘short-chain fatty acids’ or SCFAs. Those with 6-12 carbon atoms are known as ‘medium-chain fatty acids’ (MCFAs). Fatty acids consisting of 14-18 carbon atoms fall into the category of ‘long-chain fatty acids’ (LCFAs), while those possessing over 20 carbon atoms are designated ‘very long-chain fatty acids’ (VLCFAs). In terms of saturation, a fatty acid with no double bonds is labeled as saturated, whereas one with at least one double bond is termed unsaturated. Monounsaturated fatty acids possess a single double bond, while polyunsaturated variants have multiple. Polyunsaturated fatty acids can be further grouped into clusters based on the position of their first double bond, delineated as n-3, n-6, n-7, and n-9.

Non-esterified fatty acids (NEFAs), also known as free fatty acids (FFAs), are fatty acids present in the serum that are not esterified with other molecules such as glycerol or cholesterol. Elevated levels of specific FFAs in the serum, namely palmitic acid (PA) and oleic acid (OA), are intricately linked with the severity of skin inflammatory conditions. Furthermore, these particular FFAs have been demonstrated to induce a state of heightened sensitivity in dendritic cells (DCs). This sensitization leads to an augmented secretion of cytokines linked to Th1 and Th17 responses when exposed to pro-inflammatory agents (33).

Distinct subsets of T cells exhibit variable dependency on the biosynthesis of fatty acids, a process particularly crucial for the development of Th17 cells (34). Suppression of fatty acid formation not only hampers the differentiation of Th17 cells but also fosters the generation of Treg cells (35, 36). Pharmacological agents like TOFA, or the specific knockdown of acetyl-CoA carboxylase 1 (ACC1), markedly inhibit fatty acid biosynthesis in CD4+ T cells, which leads to a significant decrease in Th17 cell differentiation (34). Animal studies have substantiated this by showing that mice deficient in ACC1 possess fewer IL-17A-producing CD4+ T cells and increased expression levels of Foxp3 in CD4+ T cells. This underscores the essential role of fatty acids in modulating the balance between Th17 and Treg cells.

A diet rich in fats stimulates the formation of Th17 cells and enhances the activity of key enzymes in lipid metabolism, such as ACC1 (34). In murine models subjected to IMQ-induced psoriatic skin lesions, this type of diet intensifies the condition by elevating the levels of Vγ4+ γδ T cells capable of producing IL-17A and by augmenting the presence of activated macrophages and dendritic cells in the skin as well as nearby lymphatic channels (37).

#### 3.1.1 Polyunsaturated fatty acid

Polyunsaturated fatty acids (PUFAs) are a beneficial class of lipids crucial for maintaining optimal human health. Included in this category are both n-3 and n-6 PUFAs, which serve vital roles in neurological function as well as cellular growth and maturation.

##### 3.1.1.1 n-3 PUFA

N-3 PUFAs encompass a range of molecules such as alpha-linolenic acid (ALA), eicosapentaenoic acid (EPA), docosapentaenoic acid (DPA), and docosahexaenoic acid (DHA). These molecules appear to bear significance in the context of psoriasis. Serum concentrations of n-3 PUFAs have shown an inverse association with both the extent of skin lesions and the PASI scores in patients (38). Metabolomic assessments suggest that variations in fecal metabolites of unsaturated fats are more prominent in psoriatic arthritis patients compared to those with only psoriasis, particularly with a noted depletion in DHA levels (39–41). In 3D engineered skin models replicating psoriasis conditions, the exogenous introduction of ALA led to reduced T-cell penetration into the epidermal layer, along with a notable reduction in inflammatory markers such as CXCL1, IL-6, and IL-8 (42). Moreover, the application of a targeted FFA4 agonist resulted in substantial alleviation of IMQ-triggered psoriasis-like skin aberrations and inhibited the transition of CD4+ T cells into Th17 cells (43).

In the fat-1 mouse model, which has the unique ability to endogenously transform n-6 PUFAs into n-3 PUFAs, the prevalence of Th17 cells in the spleen is notably diminished in contrast to wild-type IMQ-treated mice. Concurrently, Treg cell population exhibited an upsurge. Upon exposure to n-3 PUFAs, Th17 cells generated reduced quantities of proinflammatory cytokines like IL-17, IL-22, and IL-23, whereas Treg cells demonstrated elevated production of anti-inflammatory molecules such as Foxp3 (44). Two mechanisms may underpin these findings. Firstly, n-3 PUFAs are metabolized into pro-resolving lipid compounds like resolvins and protectins that bolster lipid breakdown (45). Secondly, these fatty acids compete with lipoxygenase, thus causing a downregulation in the synthesis of LTB4, which has a known pro-inflammatory role (46).

DHA has been shown to mitigate the conversion of cells into the Th1/Th17 lineage (47). A DHA-derived lipophilic modulator, Resolvin E1, obstructs the migration of Th17 and γδ T17 cells instigated by LTB4. This inhibitory action could be ascribed to the selective affinity of lipoxin A4 receptor/Formyl-peptide receptor 2 (ALX/FPR2). These receptors contribute to the downregulation of cytokines connected with the IL-23/IL-17 signaling cascade and hinder key molecular pathways like MAPKs and NF-κB (48). Additionally, Maresin-1 (MaR1), another lipid entity synthesized from DHA, represses the expression of the γt (RORγt) gene in γδTCRmid+ and CD4+ T cells, which results in diminished IL-17A production (49). This evidence suggests a therapeutic advantage for n-3 PUFA in managing psoriasis.

##### 3.1.1.2 n-6 PUFA

The n-6 family of PUFAs comprises key members like linoleic acid (LA) and arachidonic acid (AA), which are notably pro-inflammatory in nature. Conversely, the n-3 PUFAs are recognized for their anti-inflammatory attributes. During instances of tissue damage or exposure to cytokines, cells liberate arachidonic acid from their membranes, setting off a cascade of enzymatic transformations. This leads to the synthesis of crucial lipid signaling molecules, specifically prostaglandins and leukotrienes. These molecules, in turn, play a vital role in elevating the differentiation and activity of Th17 cells via both straightforward and roundabout mechanisms (50).

The biosynthesis of thromboxane A2 (TxA2) is instigated by the liberation of arachidonic acid, a specific omega-6 fatty acid, from the platelet membrane. TxA2 is a potent vasoconstrictor and platelet agonist. TxA2-TxA synthase-TxA2 receptor (Tbxar2) axis is closely related to vascular dysfunction and cardiovascular disease (CVD) (51), which is one of the classic comorbidities of psoriasis. In a randomized controlled trial, low-dose aspirin improves endothelial cell health in psoriasis via platelet cyclooxygenase-1 (COX-1) inhibition, which reduces the downstream release of membrane-bound arachidonic acid and TxB2(the inactive metabolite of TxA2) (52). In studies involving the IMQ mouse model, a marked elevation of Tbxar2 mRNA expression has been observed in Vγ4+ γδ T cells, which facilitates the enhanced secretion of IL-17A in these T cells, ultimately contributing to the manifestation of skin lesions reminiscent of psoriasis. On the other hand, Tbxar2-deficient mice demonstrated diminished inflammation and a reduced population of γδ T17 cells (53). Meanwhile, Leukotriene B4 (LTB4), another lipid signaling molecule stemming from arachidonic acid, interacts with skin dendritic cells and γδ T cells through its specialized receptor BLT1. This interaction serves to stimulate their migratory activity and cytokine production, thereby exacerbating psoriatic dermatitis (54).

Induction of COX-2 expression within Th17 cells is triggered by IL-23, facilitating the synthesis of Prostaglandin E2 (PGE2), a byproduct of arachidonic acid metabolism. Upon binding to its specific receptors, EP2 and EP4, PGE2 activates various signaling cascades, including STAT3, CREB1, and NF-κB, to upregulate IL-23R expression on Th17 cells. This not only enhances the cells’ responsiveness to IL-23 but also leads to the upregulation of several inflammatory genes like IL-17A, IL-17F, and IL-18R1 within Th17 cells. Studies using IL-23-induced mouse models of psoriasis show that the targeted deletion of EP2 and EP4 receptors on T cells results in the suppression of Th17 cell accumulation and associated cutaneous inflammation (55). Therefore, targeting the PGE2-EP2/EP4 signaling axis might offer a promising avenue for the reduction of Th17 cells in psoriasis patients, positioning it as a prospective therapeutic strategy.

#### 3.1.2 Short-chain fatty acid

Short-chain fatty acids (SCFAs), including molecules like acetate, propionate, and butyrate, are the final metabolites generated by specific gut microbiota during the fermentation of dietary fiber within the colon. These SCFAs subsequently get absorbed into the bloodstream, making their way to extraintestinal tissues, including the skin. A positive modulation of regulatory T cell activity by SCFAs has been noted, offering potential therapeutic benefits for psoriasis management (56).

In murine models of dermatitis induced by IMQ, the topical administration of sodium butyrate led to noteworthy alterations in cytokine profiles. Specifically, levels of pro-inflammatory IL-17 were found to decline, while anti-inflammatory markers IL-10 and Foxp3 experienced elevated transcript levels (57). Further investigation of Treg cells, sourced from the circulatory system of individuals with psoriasis, revealed impaired suppressive function; however, this was significantly ameliorated upon sodium butyrate treatment. Lab-based assays corroborated these findings by showing that sodium butyrate not only restored the diminished Treg cell counts but also upregulated IL-10 and Foxp3 expression, while concurrently normalizing IL-17 and IL-6 levels in skin samples with psoriatic lesions. The underlying mechanism appears to be associated with epigenetic changes, specifically involving the deacetylation of histone H3 within the Treg cells (57).

Interactions between SCFAs and their receptor HCA2 are noteworthy, especially given that HCA2 expression is downregulated in lesions of psoriatic skin. In exploratory studies utilizing mice with HCA2 gene knockout (HCA2-KO), the severity of the inflammatory response to IMQ was found to be elevated, implicating a probable dysfunction in Treg cells (58). SCFAs appear to facilitate both the genesis and functional activity of Tregs. They do so by not only inducing the expression of the Foxp3 gene but also prompting dendritic cells and intestinal epithelial cells to synthesize retinoic acid and TGF-β1, respectively (59).

In patients afflicted with psoriasis, an imbalance in gut microbiota has been observed. Specifically, there is a decreased concentration of butyrate in fecal matter as compared to healthy control subjects. This imbalance leads to a reduced presence of bacteria capable of synthesizing SCFAs (60, 61). The repercussions of such a change could potentially manifest as deficiencies in Tregs. Supporting this hypothesis, murine studies using an IMQ-triggered psoriasis model revealed an inverse relationship between the serum concentrations of acetate and propionate, and cytokines IL-23/IL-27. It has been further established that oral supplementation of SCFAs can mitigate the inflammatory markers, thereby offering a glimpse into the possible utility of probiotic microbiota interventions as a psoriasis treatment strategy (62).

### 3.2 Cholesterol

In human cellular biology, cholesterol plays an indispensable role, chiefly in safeguarding the resilience and structure of cell membranes against various external factors. Notably, heightened cholesterol concentrations have been documented within the dermal tissues of those diagnosed with psoriasis (63). Such an upswing in cholesterol seems to actively contribute to the amplified release of IL-17A in individuals grappling with this dermatological ailment. Observations point towards the aggregation of certain autoantigens, encompassing both cholesterol and phospholipids, within the compromised dermal regions. It’s evident that these aggregations proactively interact with CD1b autoreactive HJ1 T cells. In related research, murine models with elevated lipid serum profiles showcased an increased secretion of IL-6 from CD1b+ dendritic cells, suggesting its role in modulating T cell orientation and heightening IL-17A output by HJ1 T cells (64). In tandem, a discernible decline in total cholesterol correlates with diminished expressions of CCL20, IL-8, and S100A7 in keratinocytes. This decline, which is instigated by IL-17A, underscores the profound link binding psoriasis to dyslipidemia (65).

Oxidized derivatives of cholesterol can be categorized as either endogenously or exogenously produced oxysterols. In instances of elevated intracellular cholesterol, indigenous enzymes synthesize endogenous oxysterols like 22-hydroxycholesterol and 27-hydroxycholesterol. Conversely, oxysterols of exogenous origin arise from the natural oxidative processes occurring in dietary cholesterol or during the preparation of cholesterol-rich foods. One such compound, 7β, 27-dihydroxycholesterol, operates as an agonist for RORγt, thus facilitating the differentiation of Th17 cells (66, 67). Recent advancements in research have shown that dietary oxysterols can exacerbate psoriasis severity by modulatingVγ2+ γδ T17 cells. They intensify γδ T17-cell-mediated inflammation via the G protein receptor (GPR183), leading to aggravated skin conditions in the IMQ-triggered mouse model of psoriasis (68). Moreover, 7-ketocholesterol (7KC), an oxysterol of exogenous nature, has the potential to worsen psoriasis-like dermatitis by hastening steatohepatitis in mice. This compound amplifies liver lipid accumulation, intensifies inflammatory cellular infiltration, and augments Th17 cell differentiation alongside the TNF signaling cascade in hepatic tissues (69). While the involvement of various lipid species in the activity of Th17 cells remains a subject of debate, there’s a growing consensus that imbalances in lipid metabolism can worsen the progression of autoimmune disorders.

### 3.3 Adipokine

Beyond its traditional role as a fat reservoir, adipose tissue possesses multifaceted endocrine attributes, releasing a broad spectrum of signaling entities. Among these, leptin, resistin, lactone, adiponectin, chemerin, retinol binding protein-4(RBP-4), and lipoprotein-associated phospholipase A2(Lp-PLA2) emerge as key mediators. The interplay of these adipokines and cytokines significantly shapes the onset and evolution of several health conditions, notably metabolic syndrome (MS) and cardiovascular anomalies. Recent findings are shedding light on the regulatory capacities of these adipokines in orchestrating immune dynamics, especially when viewed in the lens of psoriasis.

#### 3.3.1 Adiponectin

Adiponectin is a glycoprotein that exhibits a molecular weight nearing the 30 kDa range. Its structural framework can be dissected into four unique segments. Initiating with an N-terminal signal sequence, it then transitions into a non-conserved section, which exhibits high variability. Following this segment, there’s a collagen-mimetic domain characterized by 22 recurrent sequences. Culminating the structure, the C-terminal showcases a globular domain reminiscent of the C1q configuration (70, 71).

While a predominant body of studies underscores that adiponectin levels tend to be diminished in psoriasis sufferers relative to those in good health, the implications could be an enhancement of the pronouncedness of cutaneous manifestations (72, 73). Delving deeper, there is a subset of research suggesting an intriguing link: heightened IL-22 serum levels and adiponectin are positively correlated with the PASI metrics. On another note, when specifically examining the high molecular weight variants of adiponectin, it becomes evident that their concentrations are notably decreased in the serum samples drawn from individuals with psoriasis. Interestingly, there seems to be a reverse correlation between this decrease and the PASI evaluation scores (74, 75).

T-cell surfaces exhibit an abundance of receptors responsive to adiponectin, and an elevation in their expression is discernible post-antigenic stimulation. Mediating its effects via AMPK-centric pathways, adiponectin hinders the Th0 cell differentiation to both Th1 and Th17 cell lines, which results in a diminished population of IFN-γ+ T cells (76). Observations from studies involving adiponectin-lacking mice indicate an amplified cutaneous inflammatory response akin to acute psoriasis. This is characterized by a pronounced accumulation of skin-dwelling Vγ4 γδ T cells producing IL-17 and an escalated secretion of cytokines associated with Th17, namely IL-17A, IL-17F, and IL-22 (77). *In vitro* assessments underscore adiponectin’s capacity to reduce IL-17 generation in skin γδ T cells in the presence of IL-23 stimulus (77). It’s noteworthy to mention that this adipokine not only diminishes the synthesis of the cytokine IL-17 in human CD4+/CD8+ T cells (78), but also orchestrates the modulation of IL-17A mRNA expression through the AdipoR1 receptor in γδ T cells. This action further attenuates IMQ-induced skin inflammation in rodent models (79). Moreover, adiponectin alongside its analog, AdipoRon, accentuates Foxp3 expression and prompts IL-10 release, thereby adjusting the operational dynamics of human Treg cells (80). This inherent ability of adiponectin to suppress IL-17 discharge from T cells paves the way for a novel direction in the exploration of psoriasis therapeutics.

#### 3.3.2 Leptin

Produced predominantly by subcutaneous adipose tissue, leptin, a polypeptide weighing 16 kDa, arises from the ob gene situated within the chromosomal region of 7q31.3. This adipose-derived hormone carries out a myriad of physiological responsibilities, with energy regulation and immune functions at the forefront. Its association with various metabolic irregularities, such as the onset of atherosclerosis, high lipid concentration, obesity, and liver lipid accumulation, is widely recognized (81). In situations characterized by inflammation, specific cytokines, notably TNF-α, IL-6, and IL-1β, amplify leptin production by acting as potent stimulants for adipocyte activity. Within the immune spectrum, leptin plays a crucial role in directing cellular processes of both inherent and acquired immunity. Its influence spans across diverse immune cell types like granulocytes, monocytes, macrophages, dendritic entities, and T lymphocytes (82). Leptin’s interaction with the JAK2 kinase pathway triggers protein tyrosine phosphorylation events, particularly in STAT3, SHP2, and PI3K. Activated STAT3 forms dimers that are subsequently transported to the nucleus, modulating the transcriptional dynamics of several genes, with SOCS3 being of paramount importance (83). As a culmination of these processes, mitochondrial metabolic rates are augmented, thereby recalibrating energy expenditure and supervising cell proliferation and specialization.

It’s observed that individuals suffering from psoriasis tend to showcase elevated leptin concentrations, with their serum levels being markedly higher in comparison to their healthy counterparts (72, 84). A significant driver behind this increase is the IL-23/Th17 signaling mechanism. Within the immune landscape, leptin plays a central role in swaying the differentiation of CD4+ T cells towards a predominantly Th1-centric response (85). Beyond this, leptin actively aids the evolution of Th17 cells, emphasizing its versatile involvement in immune modulation (86, 87). Digging deeper, studies have spotlighted leptin’s propensity to boost the transcription activity of RORγ, an indispensable transcription agent for Th17 cellular progression (88). T cells that are bereft of operational leptin receptors display a hindered ability to morph into Th17 cells, an outcome stemming from subdued activation of STAT3 and its associated signaling trajectories (87). Delving into specific models, T-cell-specific HGK conditional knockout mice (also referred to as T-HGK cKO) with a deficiency in the leptin receptor exhibit reduced Th17 differentiation. This is coupled with improved insulin responsiveness. Such a manifestation finds its roots in the heightened activity of TRAF2/IL-6, culminating in the facilitation of IL-6/leptin-driven Th17 cellular formation and subsequent insulin resistance within adipose frameworks (89).

Within the scope of psoriasis research, leptin emerges as a notable inhibitor for Treg cells. Detailed observations from controlled *in vitro* experiments highlight leptin’s role in curbing the expansion of human Treg cells. Concurrently, it amplifies the production of an array of proinflammatory cytokines, prominently IL-6, IL-1β, IL-12, TNF-α, and IL-17 (90). An interesting avenue of investigation involves neutralizing the impact of leptin using targeted monoclonal antibodies. Such interventions lead to a surge in Treg cell proliferation, intricately connected to a downregulation of the cyclin-dependent kinase inhibitor, p27 (p27(kip1)), paired with an uptick in the phosphorylation activities of ERK1 and ERK2 (90). Diving deeper, assessments on mouse models, specifically designed to emulate autoimmune thyroiditis, indicate that impeding leptin receptors amplifies the presence of Foxp3 within the thyroid. This modulation assists in the enhanced differentiation of Treg cells and, in a contrasting manner, puts a cap on the growth trajectory of Th17 cells (91).

Emerging research underscores the multifaceted role of leptin in immunomodulation, particularly concerning its influence over diverse T cell subsets. Notably, leptin appears instrumental in promoting the proliferation of naive T cells, alongside fostering the growth of Th1 and Th17 cell subsets. This is concomitant with an enhancement in cytokine release. In stark contrast, leptin exhibits inhibitory effects on the expansion of Treg cells. Given these dynamics, it is postulated that individuals presenting with elevated leptin levels—often observed in cases of obesity—might experience altered reactivity in effector T cells and Treg cells. Such perturbations might upset the delicate balance between Th17 and Treg cells, providing a potential mechanistic link to the initiation of psoriatic conditions.

#### 3.3.3 Resistin

Resistin serves as a protein-based hormone intricately involved in processes like insulin resistance and conditions linked to obesity. This hormone is believed to obstruct the normal mechanisms of insulin signal transduction, leading to impaired cellular glucose absorption. Such interference can be a critical factor in the onset of type 2 diabetes mellitus and associated metabolic disruptions. Furthermore, emerging research data indicate that resistin also participates in the regulation of pro-inflammatory agents such as cytokines and chemokines, thereby potentially affecting the body’s immune response to infections and autoimmune conditions.

In the context of psoriasis, plasma levels of resistin are noticeably elevated when compared to healthy individuals. Moreover, there’s a significant correlation between the elevated plasma resistin and DLQI scores in psoriasis patients. Following treatment interventions, these resistin levels have been observed to decrease (92). Studies have also shown that when resistin is present in dendritic cell cultures, it may foster the expansion of regulatory T cells, commonly known as Tregs. Additionally, resistin seems to downregulate the production of cytokines like IL-6, IL-12p40, and IL-23p19 by acting upon IRF-1 within dendritic cells (93). These observations suggest that resistin could potentially serve as a counter-regulatory factor in the inflammatory processes related to psoriasis, opening up avenues for therapeutic strategies aimed at cytokine inhibition.

#### 3.3.4 Phospholipase A2

The phospholipase A2 (PLA2) group is a diverse family consisting of 11 unique members, each possessing varying substrate specificities and functions. PLA2 enzymes play a pivotal role in hydrolyzing membrane-bound phospholipids, leading to the liberation of arachidonic acid. Subsequently, this acid undergoes further metabolic transformation into eicosanoids and other bioactive lipid hormones that modulate inflammatory reactions.

In the context of psoriasis, lipidomics studies have highlighted the influence of PLA2 on alterations in ceramide levels and overall lipid metabolism within affected skin lesions (94). Heightened PLA2 expression in these lesions triggers irregular lipid metabolic pathways, fostering excessive cellular proliferation, inflammatory cytokine release, flawed keratinization, and uncontrolled cell migration (95). Gene silencing targeting PLA2s in mouse models has been shown to lessen immune activation and diminish epidermal thickening (94). T cells originating from both circulation and psoriatic skin tissue demonstrated an increased reactivity to PLA2G4D, a particular cytosolic variant of PLA2. These CD1a reactive T cells are activated by novel lipid antigens generated through exogenous PLA2, culminating in the secretion of IL-22 and IL-17A cytokines (96). Additionally, studies in animal models reveal that topically applying a lotion with PLA2G4B-siRNA can deactivate immature CD8+ T cells at cutaneous lesion sites in IMQ mice, thus mitigating the secretion of pro-inflammatory molecules like IL-17 and IL-36 (97). A newfound role for sPLA2-IIA in the regulation of gut microbiota has also been discovered. Emanating from Paneth cells, sPLA2-IIA acts as an antimicrobial agent, shaping the gut microbial landscape. This contributes to systemic inflammation, which could further impact psoriasis severity (98). Hence, therapeutic interventions blocking ceramide or sphingolipid secretion or inhibiting PLA2 activity could potentially offer targeted treatment solutions for psoriasis (99).

## 4 Regulation of amino acid metabolism on T cell in psoriasis

The amino acids involved in metabolism in the human body, including amino acids produced by the digestion and absorption of food proteins (exogenous amino acids), amino acids produced by the degradation of tissue proteins in the body, and non-essential amino acids synthesized in the body, are collectively referred to as amino acid metabolic pools. The main physiological function of amino acid metabolism in the body is to participate in the synthesis of peptides and proteins, and can also be converted into nitrogen-containing compounds such as purines, pyrimidines, and creatine. In addition, amino acids can be decomposed into α- Ketonic acids, amines, and carbon dioxide.

In patients with psoriasis, there’s a pronounced increase in amino acid metabolic activity. Through a precise metabolomics study, alterations were identified in 37 amino acids, encompassing both EAAs and BCAAs (100–102). Specifically, the levels of alanine, asparagine, aspartic acid, isoleucine, phenylalanine, ornithine, proline, aspartate, glutamate, D-glutamine, D-glutamate, glutathione, arginine, cysteine, and methionine displayed significant elevations in the context of psoriasis. It’s noteworthy that the measurement of cyclic amino acids has become an invaluable biomarker for gauging disease severity and treatment efficacy (103, 104).

### 4.1 Glutamine

Glutamine, recognized as a vital amino acid, is metabolized into α-ketoglutaric acid before its incorporation into the TCA cycle, underlining its significance in T cell operations (35, 105). Post T cell activation, there’s an augmentation in the expression of its associated glutamine transporters, enhancing glutamine assimilation and further inducing the expression of enzymes indispensable for its degradation (106). Strategies that modulate FAO or glutamine metabolism may present promising interventions for diseases steered by T cell responses (107, 108).

The metabolic process of glutamine is pivotal for IL-17 production from γδ T cells. Patients with psoriasis exhibit elevated glutamine levels and augmented expression of enzymes linked with glutamine compared to their healthy counterparts (109). During γδ T cell activation, there’s a conspicuous surge in amino acids linked to this metabolism. Hindering glutamine significantly curtails IL-17 expression from γδ T cells both in laboratory settings and real-world scenarios. This also leads to the suppression of IL-17 and IL-23/STAT3 gene pathways, thus mitigating the advancement of psoriasis in mouse models triggered by IL-23 (109). Notably, the hydrolysis of glutamine steered by Glutaminase 1 (GLS1) is anomalously proactive in both psoriasis-afflicted individuals and analogous mouse models. This hyperactivity spurs immune disequilibrium and psoriasis progression by magnifying histone H3 acetylation at the IL-17A promoter, which in turn bolsters Th17 and γδ T17 cell differentiation (110). Studies pinpoint that stalling glutamine metabolism deters the transformation of CD4+ T cells into Th1 cells but amplifies the growth and cytokine dispersion in Th17 cells. Such changes might be underpinned by GLS-incited shifts in the IL-2 and mTORC1 signaling trajectories. The subdued reaction of Th17 cells to IL-2, combined with alterations in PI3K/Akt/mTORC1 activation cues, explain the hampered differentiation of these cells in the absence of GLS (111). Conversely, when glutamine is scarce, naïve CD4+ T cells become invigorated and morph into Foxp3+ Treg cells (111). Metagenomic investigations have identified an upsurge in l-glutamate in the gut microbiota of guttate psoriasis (GP) patients. Additionally, pathway analysis elucidates that the glutamate metabolic pathway exhibits a significant interrelation with metabolites prevalent in GP. It can be inferred that l-glutamate acts as a crucial biomarker during the latter phases of the GP inflammatory reaction, influencing the interplay between DC and T cells (112). As such, glutamine metabolism emerges as a fundamental regulatory conduit for the energizing of γδ T and Th17 cells, putting forward the notion that it might offer a viable therapeutic target for ailments driven by these cells.

### 4.2 Other amino acid

Research has illuminated that the tryptophan metabolite, 5-hydroxytryptophan (5(OH)Trp), holds promise in mitigating symptoms of mouse models with psoriasis-like dermatitis. This effect is attributed to its capacity to restrain the differentiation of CD4+ T cells producing IFN-γ and IL-17A, alongside reducing the synthesis of correlated cytokines like TNF-α, IL-6, IL-17A, and IFN-γ in splenic cells. Furthermore, the presence of 5(OH)Trp weakens the expression intensity of t-bet, RORγt, and p-STAT3 in stimulated splenic cells (113).

From a metabolic vantage point, there’s a pronounced escalation in dimethylglycine and isoleucine within the bloodstream of those diagnosed with psoriasis. Both scyllitol and lysine were pinpointed to have a connection with T cell cytokine production. An amalgamation of metabolic and immune findings revealed that certain molecular features, including IL-6, IL1-ra, DMG, CCL4, IIe, Gly, and IL-8, offer diagnostic precision in differentiating newly diagnosed psoriasis patients from healthy individuals (114). Targeted metabolomics in studying amino acid variations might furnish instrumental biomarkers for early psoriasis detection and forecasting its progression.

### 4.3 Amino acid transporter

Amino acids are mainly absorbed through active transport mechanism via amino acid transporters on the cell membrane of the small intestine mucosa. The primary transport protein for L-leucine, L-type amino acid transporter (LAT), shows heightened expression in stimulated keratinocytes and immune cells including T cells, B cells, γδ T cells, macrophages, and NK cells. There’s a marked increase in LAT expression within the epidermis of individuals suffering from psoriasis. Notably, the absence of LAT1 impedes the differentiation pathways of Th1, Th2, and Th17 cells, acting independently of T cell receptor signals but via the PI3K/Akt/mTOR pathway. Moreover, curbing LAT1 or its complete absence can diminish the proliferation of γδ T cells incited by IL-23/IL-1β and curtail the secretion of IL-17/IL-22, leading to protection against IMQ-induced inflammation (115). Two transporters, Sodium Coupled Neutral Amino Acid Transporter 1 (SNAT1) and alanine-serine-cysteine transporter 2 (ASCT2), facilitate the absorption of amino acids like L-glutamine, L-alanine, and L-serine. These transporters play a crucial role in directing T cell differentiation, multiplication, and cytokine release, though their exact contribution in psoriasis remains elusive.

## 5 Discussion

**Figure 1:**
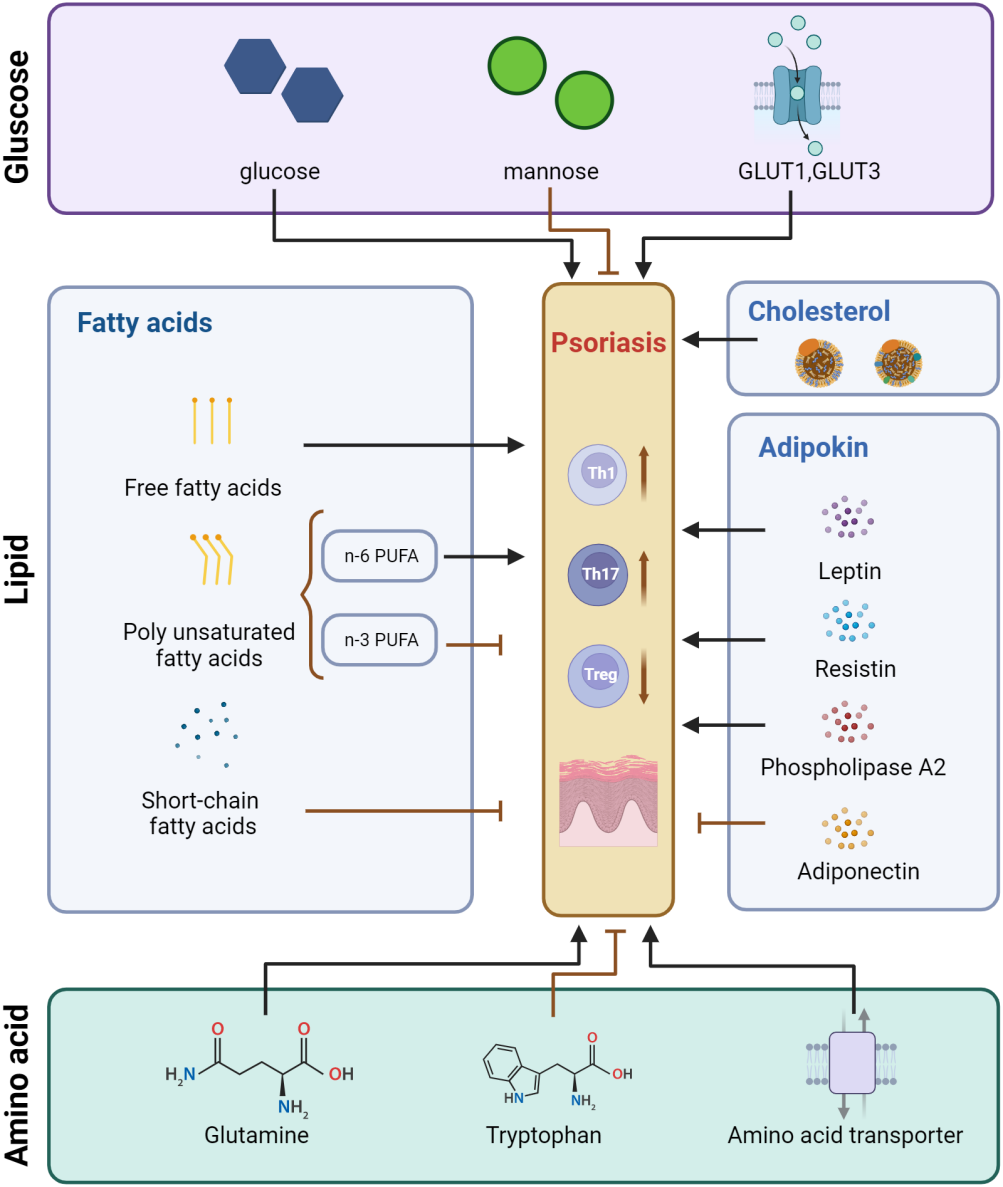
Pattern of regulatory effects of glucose metabolism, lipid metabolism, and amino acid metabolism on T cells in psoriasis. Created with BioRender.com. GLUT, Glucose transporter; PUFA, Polyunsaturated fatty acids.

In the current scientific landscape, immunometabolism has gained recognition as a rapidly growing area of research, exploring the intricate balance between metabolic regulatory processes and immune cell functionality. A case in point is psoriasis, an inflammatory skin ailment noted for its pronounced immunological markers, largely driven by T-cell mediated immune responses and often accompanied by co-occurring metabolic disturbances like metabolic syndrome. Advancements in understanding the immunometabolic controls within psoriasis pathology have verified the involvement of multiple pathways including those related to the metabolism of glucose, lipids, and amino acids, all of which contribute to modulating the T-cell driven inflammatory process (**Table 1** and **Figure 1**). Therefore, targeting the metabolic modulation of T cells opens new avenues for therapeutic interventions in psoriasis (116). These observations further clarify the link between metabolic complications, such as insulin resistance and obesity, and psoriasis. Additionally, they shed light on the higher incidence of cardiovascular metabolic comorbidities in psoriatic patients and the potential benefits of dietary changes in alleviating psoriatic skin symptoms (117, 118).

**Table 1:** Regulation of glucose metabolism, lipid metabolism, and amino acid metabolism on T cell in psoriasis.

Metabolic Pathway	Metabolite and/or target		T cell subtypes affected	Mechanism	Disorder or cells	Experimental type
	Description	Ingredient				
Glucose metabolism	Saccharide	Glucose (21)	Th17↑	Activate TGF-β and promote Th17 cell differentiation	Colitis and experimental autoimmune encephalomyelitis	Mouse model
		D-mannose (18)	γδ T↓ Th17↓	Inhibit AKT/mTOR/HIF-1α signaling pathway, ICOS, IL-17A expression and cell proliferation of γδ T cells	Psoriasis	Mouse model, cell experiment
	Transporter	GLUT1 (26)	CD4+ T↑	Increase the number of CD4+ T in psoriatic lesions	Psoriasis	Mouse model
		GLUT3 (28)	Th17↑	Regulate the pathogenic metabolic transcription of Th17 cells	Psoriasis	Mouse model, cell experiment
Lipid metabolism	Free fatty acid	PA, OA (33)	Th1/Th17↑, Treg↓	DC sensitization resulted in increased secretion of Th1/Th17 cytokines	Obesity, psoriasis	Patients, Mouse model
	n-3 PUFA	n-3 PUFA (43)	Th17↓	Inhibit CD4+ T cell differentiation into Th17 cells *in vitro*	Psoriasis	Mouse model
		ALA (42)	CD3+ T↓	Reduce T cell migration, related to p38 MAP kinase pathway	Psoriasis	Mouse model
		DHA (47)	Th1/Th17↓, Treg↑	Increase p27(kip1) and decrease Tbet, GATA-3 and RORγt. T cells co-cultured with DC-DHA express higher levels of TGFβ and Foxp3	Experimental autoimmune encephalomyelitis	Mouse model
		Resolvin E1 (48)	Th17↓ γδ T17↓	Down-regulate the expression of IL-23/IL-17 axis-related cytokines and inhibit signal transduction pathways such as MAPKs and NF-κB	Psoriasis	Mouse model
		Maresin-1 (49)	γδTCR^mid+^↓, CD4+ T↓ Th17↓	Inhibit the expression of γδTCR^mid+^ and γt (RORγt) in CD4+ T cells, and inhibit the production of IL-17A	Psoriasis	Mouse model
	n-6 PUFA	TxA2 (53)	Vγ4+ γδ T↑, γδ T17↑	Promote the secretion of IL-17 by Vγ4+γδ T and proliferation of γδ T17	Psoriasis	Mouse model
		PGE2 (55)	Th17↑	Bind to PGE2 receptors EP2 and EP4, activate STAT3, CREB1, NF-κB and other signaling pathways, and enhance the expression of IL-23R on Th17 cells; Meanwhile, the expression of Th17 inflammatory genes such as IL17a, IL17f and IL18r1 is activated	Psoriasis	Mouse model, cell experiment
	Short-chain fatty acids (SCFA)	SCFA (59)	Treg↑	Induce the expression of Treg Foxp3, promote retinoic acid or TGF-β1 to promote Treg development and function	Psoriasis	Mouse model
		Sodium butyrate (57)	Treg↑	Upregulate the expression of Treg IL-10 and Foxp3, resulting in the eacetylation of H3 histones of Treg	Psoriasis	Mouse model
	Cholesterol	Cholesterol (64)	IL-17↑	Activate CD1b autoreactive HJ1 T cells. Promote T cell polarization, and increase the production of IL-17A by HJ1 T cells	Psoriasis	Mouse model
		7β, 27-dihydroxycholesterol (61, 62)	Th17↑	Direct activation of RORγt	Th17 cell	Cell experiment, Mouse model
		Oxysterol (68)	Vγ2+ γδ T17↑, IL-17↑	Upregulate the inflammatory response mediated by γδ T17 cells, and the secretion of IL-17 by Vγ2+ γδ T17 cells increases	Psoriasis	Mouse model
		7-ketocholesterol (69)	Th17↑	Enhance Th17 cell differentiation and TNF signaling pathway in the liver	Psoriasis	Mouse model
	Adipokine	Adiponectin	Th1↓ Th17↓ IFN-γ+ T↓ γδ T↓ Treg↑	Th1/Th17 differentiation was inhibited in an AMPK-dependent manner, the number of IFN-γ+ T cells was decreased, and IL-17A, IL-17F, and IL-22 were down-regulated (76). Inhibition of skin γ δ-T cell function and IL-17 secretion (77). Promote Foxp3 expression in Treg and upregulate IL-10, which is related to phosphorylation of p38 MAPK (79, 80)	Helminth, inflammation, obesity, Psoriasis, Treg cell	Mouse model, cell experiment
		Leptin	CD4+T↑, Th17↑ Treg↓	Promote CD4+ T polarization, resulting in Th1 type immune response (85); Increased expression of RORγ promoted the differentiation of Th17 cells (86) (87–89). Inhibition of Treg proliferation and Foxp3 expression, down-regulation of p27(kip1), ERK1 and ERK2 (90, 91)	DC, Th1 cell (85), Th17 cell (87), Collagen-induced arthritis (86), Systemic lupus erythematosus (88), Insulin resistance (89), Autoimmune disease, Experimental autoimmune thyroiditis (91)	Cell experiment, Mouse model, Patients,
		Resistin (93)	Treg↓	Lead to proliferation of Treg subpopulations when CD4+ T is co-cultured with DC	DC, CD4+ Tcell	Cell experiment
		Lp-PLA2	CD1a-reactive T cell↑, CD8+ T↑ Th17↑	Induce CD1a-reactive T cell to produce IL-22 and IL-17A (96). Promote the release of inflammatory cytokines such as IL-17 and IL-36 in Th17 cells (97)	Psoriasis	Patients, cell experiment, Mouse model
Amino acid metabolism	Amino acid	Glutamine	Th1↓ γδ T↑ Th17↑ Treg↓	Glutamine deficiency can induce naive CD4+ T cells to differentiate into Foxp3+ Treg cells (111). Inhibition of CD4+ T cell differentiation to Th1 (112); Up-regulated IL-17 and IL-23/STAT3 gene signaling pathways are related to GLS-induced IL2 and PI3K/Akt/mTORC1 signaling pathways (109, 112). Enhanced histone H3 acetylation of IL17a promoter, promoting Th17 and γδ T17 cell differentiation (110)	Psoriasis Th1, Th17 cell, Th1, Treg cell	Patients, Mouse model, cell experiment
		5-hydroxytryptophan (113)	IFN-γ+ CD4+ T↓ IL-17A+ CD4+ T↓	Decrease the expression of T-BET, RORγt and p-STAT3 in activated spleen cells, inhibited the differentiation of CD4+ T cells expressing IFN-γ and IL-17A, and reduced the production of cytokines TNF-α, IL-6, IL-17A and IFN-γ	Psoriasis	Patients
	Amino acid transporter	LAT (115)	Th1↑ Th2↑ Th17↑ γδ T↑	LAT1 deficiency prevents the differentiation of Th1, Th2, and Th17 cells by activating the PI3K/Akt/mTOR pathway, and reduces the proliferation and secretion of IL-23/1β-induced γδ T cells and IL-17/22	Psoriasis	Mouse model

GLUT, Glucose transporter; PA, palmitic acid; OA, oleic acid; PUFA, Polyunsaturated fatty acids; ALA, alpha-linolenic acid; DHA, docosahexaenoic acid; TxA2, thromboxane A2; PGE2, Prostaglandin E2; SCFA, Short-chain fatty acids; Lp-PLA2, lipoprotein-associated phospholipase A2; LAT, L-type amino acid transporter.

Emerging as a burgeoning domain within systemic biology, metabolomics joins the ranks of existing disciplines such as genomics, proteomics, and transcriptomics. Leveraging high-throughput analysis of metabolites introduces an innovative methodology to elucidate underlying metabolic pathways and pinpoint novel biomarkers for psoriasis diagnosis and management. Due to the ripple effect that minor alterations in gene and protein activity can exert at the metabolic stage, metabolites become reliable indicators of an organism’s health status and phenotypic traits. Techniques commonly employed in metabolomic investigations include nuclear magnetic resonance (NMR) and high-resolution mass spectrometry (HRMS), which are utilized to assess diverse samples—ranging from skin tissues to bodily fluids like serum, plasma, and urine amino acids, as well as assorted lipid categories. The goal is to discern distinct disease-associated metabolic profiles. Pioneering non-invasive collection techniques in dermatological metabolomics, such as sweat analysis, have led to the identification of unique metabolites linked specifically to conditions like psoriasis, offering new biomarker candidates (119).

Metabolite concentrations have the potential to act as prognostic markers for concurrent medical conditions in psoriasis cases. Metabolomic profiling in psoriasis patients has shown associations between alanine, tyrosine, the unsaturation index of fatty acids, and high-density lipoprotein particles with a diminished cardiovascular threat (120). Conversely, elevated levels of glycoprotein acetyls, apolipoprotein B, and cholesterol correspond to a higher risk of cardiovascular issues. Furthermore, hydroxyalanine has emerged as a prospective marker for diagnosing psoriatic arthritis (103), while variations in levels of circulating metabolites are correlated with the activity and severity of the condition (121, 122). Plasma concentrations of specific amino acids like glutamine, threonine, ornithine, arginine, methionine, glycine, citrulline, and proline in afflicted individuals can normalize post-treatment, thus reversing most metabolic abnormalities (103). A recent comprehensive review by the BIOMAP consortium evaluated 181 studies, focusing specifically on those involving cohorts of 50 or more subjects, to collate proteomic and metabolomic markers linked with psoriasis. The study identified two noteworthy metabolomic markers, tyramine and mucic acid, as relevant to the pathogenesis of both psoriasis and its arthritis subtype (123). When comparing psoriatic arthritis (PsA) to cutaneous psoriasis (PsC), the former displayed a more pronounced upregulation of mucic acid and saturated fatty acids and a downregulation of tyramine and vitamin-associated pathways (124). Evaluating metabolite profiles in connection with the physiological state and disease activity provides an insightful methodology for gauging the effectiveness of psoriasis treatments, thereby adding clinical value to personalized healthcare plans.

Modifying metabolic pathways emerges as a novel strategy for psoriasis intervention (125). Dietary modifications can beneficially affect a patient’s life quality by ameliorating skin lesions and reducing the risk of additional health issues. In psoriasis-like dermatitis models induced by IMQ, adopting a Western diet for a brief four-week period has been shown to boost the population of γδ T cells producing IL-17A, and to elevate the activity levels of the IL-23 receptor (126). However, the severity of dermatitis symptoms does not worsen with a high-fat but low-sugar diet, highlighting the exacerbating role of sugar content in Western dietary habits (127). For those dealing with both psoriasis and weight issues, a low-calorie diet comes highly recommended. Incorporating EPA and DHA from fish oil and corn oil can offer symptomatic relief in psoriasis cases (128). A thorough systematic review conducted by Chen et al., covering 18 randomized controlled trials, corroborated that fish oil and its derivatives can attenuate risk factors associated with obesity, cardiovascular ailments, and metabolic imbalances in psoriasis patients, while also modulating various inflammatory markers (129). It is advisable for psoriasis sufferers to limit saturated fats and to opt for anti-inflammatory omega-3 polyunsaturated fats, while also reducing the intake of omega-6 acids (130). Moreover, a ketogenic diet, characterized by lower carbohydrate intake and relatively increased consumption of fats and proteins, holds potential in rectifying metabolic and inflammatory imbalances related to psoriasis (102). In essence, nutritional approaches significantly influence clinical, metabolic, and inflammatory markers in individuals with psoriasis.

In addition to glucose metabolism, lipid metabolism and amino acid metabolites, oxidative stress, iron metabolism, nitric oxide metabolism and other processes and metabolites are also closely related to psoriatic T cells, and then affect the progression of psoriasis (131–133). Increased ROS levels can activate NF-κB and MAPK, and then activate psoriasis Th1 and Th17 cells (134). Iron ion is a key factor in the production of ROS via enzymatic or non-enzymatic reactions and participates in lipid peroxidation. ROS and ferrous ions are increased in the epidermis of patients with psoriasis vulgaris, while glutathione peroxidases 4 (GPX4) is decreased (135, 136). The latter is a GSH-dependent monomeric enzyme that plays a role in protecting Treg cells from iron-dependent lipid peroxidation-mediated death, whose absence disrupts Treg cell mitochondrial homeostasis and increases IL-1β production (137). Nitric oxide synthase 2/inducible nitric oxide synthase (NOS2/iNOS) produces nitrous oxide subsequent to the stimulation of pro-inflammatory cytokines, which is an innate immune marker and has great significance in the diagnosis of psoriasis (133). Studies have found that Nos2-induced NO promotes mannose-induced Ps and PsA models (MIP) in mice, and Nos2-dependent IL-1α is essential for arthritis development by promoting IL-17 production of innate lymphoid cells (138). Furthermore, targeting IL-35-induced nitric oxide synthase may serve as a new therapeutic strategy for patients with chronic psoriasis (133).

To sum up, metabolomic studies are enriching our diagnostic toolkit with critical biomarkers that can guide both the identification and management of psoriasis. These studies are not only deepening our insight into the etiology of psoriasis and related metabolic imbalances but also offering robust indicators for evaluating the course of the disease and the efficacy of treatment protocols. As we continue to unravel the complexities of how skin metabolism interacts with T-cell immune responses and inflammatory metabolic pathways, focusing therapeutic efforts on specific subsets of T cells in psoriasis, especially Th17 cells, may pave the way for more personalized medical interventions. This could be particularly beneficial in pinpointing patients who may benefit from close surveillance or targeted treatment options.

## Author contributions

RS: Conceptualization, Investigation, Validation, Writing – original draft, Writing – review & editing. SZ: Data curation, Writing – original draft. JZ: Data curation, Writing – review & editing. MC: Methodology, Writing – review & editing. SP: Project administration, Supervision, Validation, Writing – review & editing.
